# Supplementary material for: The Impact of COVID-19 on the Working Equid Community: Responses from 1530 Individuals Accessing NGO Support in 14 Low- and Middle-Income Countries
Source: Animals (Basel). 2021 May 11;11(5):1363. doi: 10.3390/ani11051363 (PMC8151231; doi:10.3390/ani11051363)
Supplement: Supplementary file 1 [file animals-11-01363-s001.zip › animals-1187116-supplementary.pdf]

Supplementary Items

Survey Overview

The survey was designed as an online survey (SmartSurvey™) with 38 questions. Details of the question type are provided in Table S1 and details of question text and available response are provided in Table S2.

Table S1: Survey question types.

Question Type	Number of Questions
Checkbox – single answer	20
Checkbox – single answer *required	2
Checkbox – multi answer	5
Open	4
Open *required	1
Open- Matrix of text questions	1
Open- Multiple textboxes	1
Matrix of choices (one answer per row)	1
Matrix of choices (multiple answers per row)	2

Table S2: Survey questions analysed.

Question number	Question type	Question text	Answer choices
1	Checkbox – single answer *required	1. Participant Consent*:<br>• I have read/listened to the Participant Information<br>• I understand the purpose of the research and my role in it<br>• I understand I may stop the interview at any point<br>• I understand I have the right to withdraw from the project at any point<br>• I understand that the study is anonymous and no personal information will be collected, and if results are published I will not be identified<br>• I understand that the information collected will be	Yes<br>No

		stored by World Horse Welfare and only accessible by researchers<br>• I understand I can contact the researchers/organisations if I wish to gain more information regarding this study<br><br>I consent to taking part in this survey.	
2	Checkbox – single answer	How are you conducting this survey?	Face to Face<br>By telephone<br>Online<br>Other (please specify)
3	Checkbox – single answer *required	Country	Costa Rica<br>Colombia<br>Guatemala<br>Honduras<br>Nicaragua<br>Panama<br>Mexico<br>Haiti<br>South Africa<br>Zimbabwe<br>Lesotho<br>Senegal<br>Nepal<br>Nepal (India)<br>Cambodia
5	Open	Number of family members in household	Open answer
6	Open-Matrix of text questions	Number / type of equines owned Currently:	Horse- open answer<br>Donkey- open answer<br>Mule- open answer
		Number / type of equines owned before Covid-19 Pandemic (e.g. January 2020)	
7	Open	If the number has changed please comment why/what happened to equid(s)?	Open answer
8	Checkbox – single answer	Has there been a change in your equid's workload in since Covid-19 pandemic ? (e.g. January 2020)	Working more<br>Working same<br>Working less<br>Not working

9	Checkbox – multi answer	If your equid's workload has changed, why? (select all that apply)	Government enforcement/national restrictions<br>Local enforcement/regulations<br>Change in demand<br>Change in financial situation<br>Other (please specify)
11	Checkbox – single answer	Has your monthly income from your working equid changed since Covid-19 pandemic? (e.g. January 2020)	Decreased<br>Stayed the same<br>Increased
12	Matrix of choices (multiple answers per row)	What type of work is your equid doing? (select all that apply)- currently	Ranching/herding<br>Crop transport<br>Water transport<br>People transport<br>Ploughing<br>Racing<br>Tourism<br>Freight transport<br>Other transport<br>Comments
		What type of work is your equid doing? (select all that apply)- Before Covid-19 Pandemic (e.g. January 2020)	Ranching/herding<br>Crop transport<br>Water transport<br>People transport<br>Ploughing<br>Racing<br>Tourism<br>Freight transport<br>Other transport<br>Comments
13	Checkbox – single answer	If you sell your equid now would you expect to sell it for more or less than what you could sell it for in 2019 (e.g. January 2020)?	More<br>Same<br>Less<br>Don't know<br>Give examples if possible.

14	Checkbox – single answer	Has the total cost of owning/upkeep of your equid changed since Covid-19 (e.g. January 2020)?	Increased<br>Same<br>Decreased<br>Don't know
16	Checkbox – single answer	Has the cost of using these services changed?	Increased<br>Same<br>Decreased<br>Don't know
17	Checkbox – single answer	Has the availability of using these services changed?<br>Increased	Increased<br>Same<br>Decreased<br>Don't know
18	Checkbox – single answer	Have you seen any changes in the health of your equid since Covid-19 pandemic? (e.g. January 2020)	Improved<br>Same<br>Deteriorated<br>Don't know<br>Other (please specify)
20	Checkbox – single answer	How is happy/well is your equid currently?	Very poor<br>Poor<br>Good<br>Very good<br>Don't know<br>Comments
22	Checkbox – single answer	Have you seen any new health problems in your equid since Covid-19 pandemic? (e.g. January 2020)	Yes<br>No<br>Don't know<br>If yes, please give examples
23	Checkbox – single answer	Has the weight/body condition of your equid changed since Covid-19 pandemic? (e.g. January 2020)	Increased weight<br>Same<br>Decreased weight<br>Don't know
24	Checkbox – single answer	Have your total household monthly expenses/outgoings changed since the start of the pandemic? (e.g. January 2020)	Decreased<br>Stayed the same<br>Increased<br>If changed, why?

25	Checkbox – single answer	Has your total household income changed since the Covid-19 pandemic? (e.g. January 2020)	Decreased<br>Stayed the same<br>Increased<br>If changed, why?
26	Checkbox – multi answer	Have you had to supplement your income in other ways, if so how?	Family<br>Friends<br>Government<br>NGOs<br>Bank<br>Money lender<br>Extra jobs<br>Aid support/remittances<br>None of the above<br>Comments
27	Matrix of choices (one answer per row)	In your opinion is your financial situation better than others in the same community that do not have a working equid? – Currently	Better<br>Same<br>Worse<br>Don't know
		In your opinion is your financial situation better than others in the same community that do not have a working equid? – Before Covid-19 Pandemic (e.g. January 2020)	Better<br>Same<br>Worse<br>Don't know
28	Checkbox – multi answer	What type of support schemes are currently available to you?	Financial<br>Governmental assistance<br>NGO support<br>World Horse Welfare feed relief<br>Don't know<br>Other (please specify)
29	Checkbox – single answer	How easy are these schemes to access?	Very easy<br>Easy<br>Neither easy nor difficult<br>Difficult<br>Very difficult<br>Don't know
30	Checkbox – single answer	Have you used any of these schemes?	Yes<br>No<br>Other (please specify)
31	Checkbox – single answer	If so, how beneficial did you find them?	Not beneficial<br>Not very beneficial<br>Beneficial<br>Very beneficial<br>Don't know

33	Checkbox – single answer	Are there any support schemes to help directly with animal health?	Yes<br>No<br>Don't know<br>If Yes, please specify
34	Checkbox – single answer	Would you feel comfortable having outsiders entering your community?	Yes<br>No<br>Don't know<br>Comments
35	Checkbox – multi answer	What aid would you find most beneficial? (please select all that apply)	Vet support<br>Feed<br>Equid first aid<br>Shoeing<br>Vaccinations<br>Anthelmintics/dewormer<br>Financial support<br>Other (please specify)
36	Checkbox – single answer	What is your current level of anxiety in regards to your own economy/livelihood?	Severe anxiety<br>Moderate anxiety<br>Mild anxiety<br>No anxiety
37	Open	Can you mention a positive aspect that has occurred due to Covid-19?	Open answer

Table S3: Survey questions not analysed.

Question number	Question type	Question text	Answer choices
4	Open *required	Name of Community	Open answer
10	Open- Multiple textboxes	What proportion of your monthly income is derived directly from your working equid? (approximately - 1-100%)- Currently	Open answer
		What proportion of your monthly income is derived directly from your working equid? (approximately - 1-100%)- Before Covid-19 Pandemic (e.g. January 2020)	Open answer

15	Matrix of choices (multiple answers per row)	Do you use any of these services? – currently	Farrier<br>Vet<br>Saddler/harness maker<br><br>Paravets/ technicians /<br>Agrovet/ dewormer/ medications<br>Animal welfare assistance<br><br>None of these<br>Other (please specify)
		Do you use any of these services? – Before Covid-19 Pandemic (e.g. January 2020)	Farrier<br>Vet<br>Saddler/harness maker<br><br>Paravets/ technicians /<br>Agrovet/ dewormer/ medications<br>Animal welfare assistance<br><br>None of these<br>Other (please specify)
19	Open	If changed please give examples?	Open answer
21	Checkbox – single answer	Has there been a change in severity/frequency of any pre-existing health problems in your equid since the Covid-19 pandemic? (e.g. January 2020)	Improved<br>Same<br>Deteriorated<br>Don't know<br>No pre-existing health problems<br>If yes, please give examples
32	Checkbox – multi answer	What are the most significant/immediate issues for you and your equid(s) please select all that apply	Feed<br>Vet support<br>Stabling<br>First aid<br>Lack of income<br>Your personal health<br>Other (please specify)
38	Open	Is there anything else you would like to mention/ask?	Open answer

Table S4: Country context information based on anecdotal reports from project start

Country	COVID-19 situation	Seasonal considerations	Adverse events
Lesotho	"Purple" level restrictions [mid-level restrictions]. Schools and restaurants open. Sports with no spectators. **<100 people allowed to meet outside**.	**Seasonal impact highly prominent**. Country has just left winter season: nutritionally, **equids underfed due to poor pastures**, owners do not always supplement feed. Rains have increased past few weeks so vegetation will begin to improve.	COVID-19
Senegal	Initially, COVID-19 situation was improving over the past 3 months. Between March and July, there were strong restriction measures. Now, all activities have resumed with barrier measures: social distances and masks in certain places. Many feel in the "post-Covid" situation, the situation has changed drastically for owners. **Second wave of COVID-19 started during data-collection period: 5th December, impacting number of respondents. Gathering of responses over phone from 15th December.**	**75% of owners are seasonal workers**, during the rainy season they leave for agriculture. There are no major trips as it is the end of winter.	COVID-19
Zimbabwe	Nationwide lockdown from end March-August – strong restrictions, now "everything returned to normal" but increasing cases over 3 months. Still required: social distancing, sanitisation, masks in public, <100 outside, curfew. Schools opened 9th Nov – spike in cases. In communities – minimal effects – **many disregarding the measures – situation has drastically changed from when the owners were unable to work**.	Within the project area there has **been no vegetation for grazing and the condition of the donkeys has been very poor** such that in some communities the equids are no longer working. During the second week of December 2020 some rains have been received and owners have been busy gathering their donkeys preparing to begin ploughing.	COVID-19<br>**Droughts**
South Africa	**Second wave** of infections – high where the cart horse owners live. **Most people do not adhere to the basic rules of wearing a mask, sanitising and social distancing** and must be constantly reminded. Recent announcement: curfews, beach and recreational area closures. There is a lot of concern about people travelling to be with family as at this time of the year (the festive	It is summer, but a very mild one to date. No impact of the equine owners. Business as usual**. The summer months the horses usually work longer hours due to longer daylight hours.**	COVID-19

	season), there is a lot of movement from province to province.		
Nepal	First country-wide lockdown March-July. Re-opening of business caused rise of cases, second lockdown August. Every day 700-800 new cases have emerged in Nepal, **the real picture of COVID-19 is unknown due to poor testing** (difficulties with government and private testing capacities and effects on insurance). All restrictions now removed, "life as normal". India/Nepal border – restrictions. More cases in Kathmandu vs countryside. COVID-19 impacted equines/owners, the government-imposed lockdown in the brick production season and still the brick kilns across the country are not opened in full phase. **The closure of the brick kilns in the earlier season in beginning of 2020 made it difficult for equine owners to get income** which impacted owners and equines.	Most of the equine owners do work in brick kiln during the **brick kiln season from November to June and get income for their entire livelihood for a year**. The low temperature and fog in the area have been observed from last week. Chilly weather and cold breeze during the **winter season always adversely effects the daily life of many** people in Banke and Kailali. Last year the death of people was observed in Kailali and Banke because of low winter temperatures.	COVID-19<br>Ongoing **political instability** – mass agitation.
Cambodia	Low case rate, but semi lockdown from April, preventing travel between provinces closures of schools, universities, gyms, entertainment centres – reopened September. Hygiene measures: masks, sanitisation, social distancing. Small outbreak due to international travel beginning of November, no group gathering and closure of business until 23rd November (affecting cities). **Impact on equine owners throughout pandemic: transporting goods to market and tourism**. During the month of November when the survey started to be conducted, the activities of the pony owners return to normal (free movement or free travelling without restriction).	**Flooding** (and pandemic) has reduced usual clinic rotation frequency. No more flooding issues, entering dry season.	COVID-19<br>**African Horse Sickness** outbreak in February in Thailand (preventative measure implemented)<br>**Floods**
Colombia	Risk of COVID-19 is high. During the weekends there is a curfew. You cannot leave from a certain time; it all depends on how the infections develop during the week.	Consequences of the **hurricane, there are many floods and landslides**. The start of winter has increased the contagion.	COVID-19<br>**Hurricane**

	**There are departments where they are strict, others not so much.** Even people without masks.		
Nicaragua	People affected by COVID-19 have decreased**. However, many do not follow biosafety measures. There is no quarantine**, things are “normal” (in quotation marks).	Environmentally, the country has just been hit by **two hurricanes** and there is a lot of wind and rain. There is a lack of grass due to it being the summer. Equids are usually seen in poorer condition at this time of year due to these factors.	COVID-19<br>**Hurricane**
Costa Rica	Currently the infection rate has increased in Costa Rica, getting around 1000 people infected per day. **Medical centres are collapsing making even less opportunity for patient care**. Since Christmas is so close, people are very relaxed and not following correct distancing measures.	There is no adverse weather, everything is normal for the season.	COVID-19
Panama	Cases are rising with a regrowth and is **reaching further out communities**. There is curfew, ban on sale of alcoholic beverages, quarantine of communities with high infection rates, possibly from 20th Dec closure of non-essential shops. Christmas and New Years Day may not be complete quarantine.	The climate in Panama is not very defined as we are ending the **phenomenon “La Niña”**, it rains at times of the day and then the sun rises for other days. The owners have chosen to let their horses rest while the weather changes.	COVID-19<br>**Heavy rains** preventing visits to some communities
Guatemala	Cases being reported, most commonly mild respiratory problem, only small numbers dying (due to co-morbidities). **Many patients fear going to diagnostic centres and hospitals, so stay at home to recover.** Currently social distancing, masks and hand disinfectants required. The equine owners’ economy has been effected – there is none coming into the community, only remittance income. Many were working away from their communities but had to return back. **Being unable to travel to neighbouring communities or towns to sell goods has affected income.**		COVID-19
Mexico	Currently high infection rates, government taking more drastic measures in the city. Restrictions continue in the state, no mass events, educational institutions remain closed, planned re-opening August 2021. **Markets are**	Rainy season has not favoured crops, owners have to buy alfalfa. Owners currently harvesting.	COVID-19<br>**Roadblocks**

	**closed, owners cannot sell products and income considerably affected. Some have had to sell their animals to cope with the situation.**		
Haiti	Government have stopped giving daily case updates, **no reports on current COVID-19 situation**. It is present but not hearing about many being hospitalised or dying with the virus**. Economy decrease due to many factors**, however there **isn't anything abnormal noticed due to COVID-19.**		COVID-19
Honduras	Cases have been increases since businesses are re-opening. Most businesses are open, including busses and taxis (since strikes) with biosecurity measures including masks. Curfews in place.	In November Honduras was hit by **2 Hurricanes** called ETA and IOTA, people in some areas were affected **with much raining, flooding and lost some belongings.**	COVID-19<br>**Hurricanes**

Summary of feedback from project staff (S5):

Most staff found using a tablet computer with survey link worked well for data collection and had no technically difficulties (10/14), however some did not use tablets (2/14) or had problems with the offline link (3/14). Most found that participants were easy and willing (12/14), however some found it difficult due to the time of year (2/14) amongst other reasons. Examples of comments: "Newer participants to project trickier, but established owners in project no problem", "Not easy: worried about Covid, do not want to meet, time constraints, shy, no ideas/replies, never done a survey like this before", "2 or 3 days in advance the community leader was contacted, so this was easy". The majority conducted surveys face-to-face (Figure 2), some adding it was better, faster, fewer interruptions or no problems with signal, however, two projects said there was no difference between face-to-face or over phone. Comments included: "Most owners don't have a cell phone", "Worry about COVID-19 with face to face" and "More time spent travelling with face to face". Some projects considered their situation to be as expected (5/14), with some reporting that they found that owners were struggling more than expected (3/14), with some differences between project areas/communities and one project reporting "equines were in good condition, better than expected".

Most project staff said that the questions worked well (10/14), with comments on some questions being too similar (3/14), or sometimes owners not understanding wording/translation (3/14). There were several comments (7/14) on how individual questions could be improved and there was variation between countries. For example, in one African country "Numbers of people in the household the trickiest question due to socio-cultural reasons", which was unique to this project and some other projects found "Owners did not want to share personal issues e.g. income". For improving the survey for the next round, the top comment was that the survey needs to be shorter, more focussed with similar questions combined (7/14), that they need more time to complete the surveys e.g. a longer data collection period to meet the targets (4/14), however some wanted more questions e.g. mentioning specific health problems faced the equid (listing options) (2/14). The main barriers to carrying out the survey were a lack of time (6/14), Covid-19 (3/14) and long distances to get to communities (2/14). Half of the projects reported they would not require any further training for future rounds; however, it will be provided, and will be required if there are changes to the survey schedule. Comments included "Need better understanding of each question's purpose", "Coaching received was well done" and "Will always take more training if available to increase knowledge.".

All the project staff reported that undertaking the survey has been a useful activity for their projects. The main comments included: "The results are going to be used to make decisions and actions", "Owners can see we are concerned for their wellbeing", "We can see where the needs are" and "We now know about the economics and livelihoods of owners."
